# Cardiopulmonary Function and Aerobic Exercise in Parkinson's: A Systematic Review of the Literature

**DOI:** 10.1002/mdc3.13011

**Published:** 2020-07-25

**Authors:** Aseel Aburub, Sean J. Ledger, Julius Sim, Susan M. Hunter

**Affiliations:** ^1^ Faculty of Medicine and Health Sciences School of Allied Health Professions, Keele University Keele United Kingdom; ^2^ School of Health, Medical and Applied Sciences, Central Queensland University Rockhampton Queensland Australia; ^3^ Faculty of Medicine and Health Sciences School of Allied Health Professions and School of Primary, Community and Social Care, Keele University Keele United Kingdom; ^4^ Faculty of Medicine and Health Sciences, School of Allied Health Professions Keele University Keele United Kingdom

**Keywords:** pulmonary function, spirometry, cardiopulmonary exercise test, walking economy, aerobic exercise, Parkinson's

## Abstract

**Background:**

Cardiorespiratory impairments are considered the main cause of mortality in the late stages of Parkinson's. Aerobic exercise has been shown to improve pulmonary function in asthmatic patients and in healthy people. However, effects of aerobic exercise on cardiopulmonary function in people with Parkinson's have not been investigated. Therefore, this study aimed to review the effects of aerobic exercise on cardiopulmonary function in people with Parkinson's.

**Methods:**

A systematic search was conducted using MEDLINE, AMED, CINHAL Plus, and relevant associated keywords, from January 1970 to January 2020. Inclusion criteria for the studies were: aerobic exercise as part of the intervention, pulmonary function test, and/or cardiopulmonary exercise test as outcome measures.

**Results:**

In total, 329 citations were identified from the search, of which nine were included in this review. In general, aerobic exercise was found to have positive effects on cardiac function for people with Parkinson's, but there is a lack of studies on the effects of aerobic exercise on pulmonary function.

**Conclusion:**

People with early stages of Parkinson's may experience positive effects of aerobic exercise on cardiac fitness. Further research is needed in this area, particularly into the effects of aerobic exercise on pulmonary function in early stages of the disease.

Parkinson's is the most common age‐related, neurodegenerative motor disorder with unknown cause.^1^ Respiratory problems, which can present as either a restrictive or an obstructive pattern, are the main cause of morbidity and mortality in the end stages of Parkinson's.^2^ Restrictive pulmonary disease, or restrictive pattern, is characterized by decreased lung volumes, increased work of breathing, and inadequate ventilation and oxygenation, and includes pulmonary fibrosis, pneumonia, and pulmonary edema.^2^ Obstructive pulmonary disease is generally characterized by inflamed and collapsible airways and impediment to airflow during exhalation, which is commonly exhibited in asthma, chronic bronchitis, and emphysema.^2^ Most people with Parkinson's (PwP) do not report respiratory problems in the early stages of the disease, although evidence from cross‐sectional studies has highlighted abnormalities in pulmonary function.^3^, ^4^, ^5^, ^6^ These might be because of the low levels of physical activity caused by motor symptoms, during which respiratory function is unchallenged, and consequently, respiratory impairment or complaints are not manifest.^6^

Aerobic exercise refers to the use of oxygen to adequately meet energy demands during physical exercise.^7^ In the general population, heart rate (HR) and respiratory rate increase during aerobic exercise to fulfill demands of the exercising skeletal muscles.^7^ According to the new UK Chief Medical Officer's Physical Activity Guidelines,^8^ older adults should aim to accumulate at least 150 minutes per week of moderate‐intensity aerobic activity to gain health benefits, including maintenance of good physical and mental health, wellbeing, and social functioning. Additionally, it has been found that regular aerobic exercise, such as walking, cycling, or swimming, of 30–60 minutes three times per week can decrease blood pressure, improve oxygen consumption, and decrease shortness of breath in the general population.^9^ However, these effects have not been widely explored in PwP.

A number of studies^10^, ^11^ have investigated the effects of exercise and physical activity on balance, mobility, quality of life, and cognitive function in PwP. However, there has been limited research on cardiopulmonary responses to exercise in PwP. Therefore, this review evaluated the published literature to answer the following research question: what are the effects of aerobic training on cardiopulmonary function in PwP?

## Methods

### Purpose

The study objective was to review the reported effects of aerobic exercise on cardiopulmonary function and walking economy, and the effects of interventions that might improve cardiopulmonary function in PwP. To achieve this, the primary outcomes are defined as pulmonary function test (PFT) variables including forced expiratory volume in 1 second (FEV_1_) and forced vital capacity (FVC) and FEV_1_/FVC and cardiopulmonary exercise test (CPET) variables including oxygen uptake at maximal exertion (V˙O_2max_) and oxygen uptake at peak exertion (V˙O_2peak_). Secondary outcome measures include maximum HR (HR_max_) and peak HR (HR_peak_), CPET duration, blood pressure pre‐ and post‐CPET, and walking economy. The study aimed to conduct a meta‐analysis for the primary outcome measures where feasible. If conducting meta‐analysis was not possible, the study aimed to conduct a narrative synthesis.

### Design

The study was designed to provide a systematic review with quality assessment and narrative synthesis of relevant published literature.

### Search Strategy

A search was conducted through EBSCO using the following electronic databases: MEDLINE, AMED, and CINHAL Plus. The search was limited to full‐text English‐language articles and excluded conference abstracts. The selected databases were chosen because of the likely availability of Parkinson's physiotherapy‐ and exercise‐related articles in these databases. The databases were searched for studies published between 1st January 1970 to 1st January 2020, with results of the searches managed using Endnote Version X7 (Clarivate Analytics, Philadelphia, PA). Table 1 summarizes the combinations of keywords included in the search strategies.

**TABLE 1:** *Summary of search strategy and records of findings*

	Search Strategy
Search strategy	1 = Parkinson's disease 2 = Parkinson's 3 = 1 OR 2 4 = Aerobic exercise 5 = Exercise 6 = Physical activity 7 = Training 8 = 4 OR 5 OR 6 OR 7 9 = Pulmonary function 10 = Respiratory function 11 = Spirometry 12 = Spirometer 13 = Cardiovascular response 14 = Cardiopulmonary response 15 = Exercise stress test 16 = Exercise test 17 = Cardiopulmonary exercise test 18 = Graded exercise test 19 = Walking economy 20 = 9 OR 10 OR 11 OR 12 OR 13 OR 14 OR 15 OR 16 OR 17 OR 18 21 = 3 AND 8 AND 20
Records retrieved	329
Records included	9

Randomized controlled trials, controlled clinical trials, and controlled quasi‐experimental studies investigating the effects of aerobic exercise on cardiopulmonary function in PwP were included. Studies that did not include either a CPET or a PFT were excluded. Pre‐clinical studies (animal model studies) were excluded. Single‐group, uncontrolled, and single‐case studies were excluded.

### Study Selection

Following the search and subsequent removal of duplicates, titles and abstracts were screened by two researchers (AA, SMH) for relevance. Full texts of relevant studies were then screened for eligibility against inclusion and exclusion criteria.

### Data Extraction

The following data were extracted from the included studies and presented in a table: age, sex, disease severity, and sample size. Exercise intervention mode, intensity, duration, and frequency were noted.

### Quality Assessment of the Included Studies

Quality assessment of included trials was performed using the PEDro Scale, a valid and reliable tool for assessment of quality of interventional studies specifically related to physical therapy interventions.^12^, ^13^ The PEDro scale contains 11 items (see Table 2), and studies are awarded between 0 and 10 points, depending on the number of criteria they meet (the first item is not used to calculate the summary score). Studies with scores of four points or more were classified as “high‐quality”, whereas studies with three points or fewer were classified as “low‐quality”.^12^ PEDro scores for the studies were not used as an inclusion or exclusion criterion, but as a basis for best‐evidence synthesis and to determine the strengths and weaknesses of each study.

**TABLE 2:** Quality assessment for the included studies^a^

	1^b^	2^c^	3^d^	4^e^	5^f^	6^g^	7^h^	8^i^	9^j^	10^k^	11^l^	Total PEDro Score
Bergen et al.^14^	1	0	0	1	0	0	0	1	1	1	1	5
Bridgewater and Sharpe^18^	1	1	1	1	0	0	1	1	1	1	1	8
Burini et al.^16^	1	1	1	1	0	0	1	1	1	1	1	8
Corbianco et al.^15^	1	1	1	1	0	0	0	1	1	1	1	7
Fernández‐del‐Olmo et al.^22^	1	1	0	1	0	0	0	1	1	1	1	6
Mavrommati et al.^17^	1	1	1	1	0	0	1	1	1	1	1	8
Ridgel et al.^20^	1	1	1	1	0	0	1	1	1	1	1	8
Schenkman et al.^21^	1	1	1	1	0	0	1	1	1	1	1	8
Shulman et al.^19^	1	1	1	1	0	0	1	1	1	1	1	8

^a^ PEDro scores for the studies that investigated the effects of aerobic training on cardiopulmonary function in people with Parkinson's.

^b^ Eligibility criteria were specified.

^c^ Subjects were randomly allocated to groups.

^d^ Allocation was concealed.

^e^ The groups were similar at baseline regarding prognostic indicators.

^f^ There was blinding of all subjects.

^g^ There was blinding of all therapists who administered the therapy.

^h^ There was blinding of all assessors who measured at least one key outcome.

^i^ Measures of at least one key outcome were obtained from more than 85% of the subjects.

^j^ All subjects for whom outcome measures were available received the treatment or control condition as allocated.

^k^ The results of between‐group statistical comparisons are reported for at least one key outcome.

^l^ Point measures and measures of variability for at least one key outcome were reported.

## Results

**FIG. 1:**
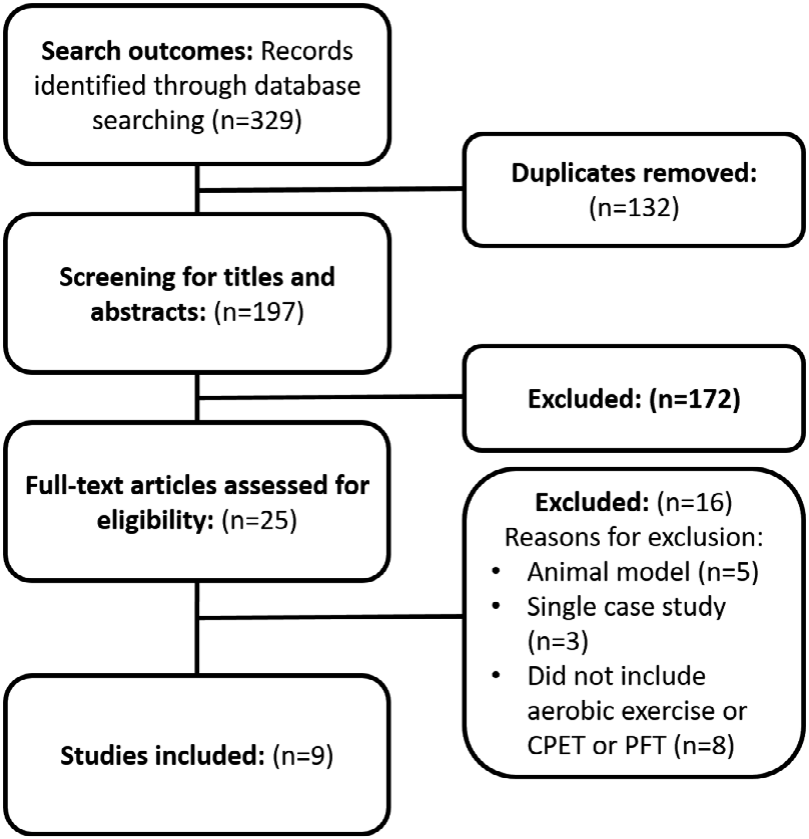
Flow‐chart of the search outcomes. PFT, pulmonary function test; CPET, cardiopulmonary exercise test.

The systematic search identified 329 citations, of which 132 were duplicates. Consequently, 197 citations were screened from titles and abstracts and 172 were considered not to be relevant. Of the 25 remaining studies, 16 were excluded because they did not include aerobic exercise or CPET or PFT, or they were animal model studies or single‐case studies. Consequently, nine studies were included in the review: one was a non‐randomized controlled pilot study^14^ and eight were randomized controlled trials.^15^, ^16^, ^17^, ^18^, ^19^, ^20^, ^21^, ^22^ Figure 1 represents the findings of the search.

### Quality Assessment

Because of the nature of exercise interventions, no studies included blinding of subjects or investigators to the intervention allocation; therefore, no points were awarded on the PEDro Scale for items 5 and 6. Table 2 shows the PEDro quality assessment scores for the included studies. Scores ranged from 5^14^ to 8.^16^, ^17^, ^18^, ^19^, ^20^, ^21^

To assess the feasibility of running meta‐analysis, data were extracted and summarized in Table 3 and focused on: pulmonary function test variables; the protocols used for CPET; mode of the test: treadmill or cycle test; CPET test primary outcomes (V˙O_2max_ and V˙O_2peak_); secondary outcomes including HR_max_; HR_peak_; CPET test duration; blood pressure (BP) pre‐ and post‐CPET; and walking economy. None of the studies investigated the effects of aerobic exercise on pulmonary function; therefore, no data related to FEV_1_, FVC, and FEV_1_/FVC could be reported. Meta‐analysis of data was not undertaken owing to the heterogeneity of the studies, specifically: inclusion/exclusion criteria, exercise test protocol, mode of exercise test (cycle or treadmill), exercise intensity (maximum or sub‐maximum), physiological outcomes (HR, V˙O_2peak_, V˙O_2max_), and systolic or diastolic BP. Instead, a narrative review was conducted. Furthermore, it was not possible to calculate effect sizes from the data provided in the published papers. Authors were contacted by email to request the additional relevant data, but none responded.

**TABLE 3:** *Summary of studies that investigated the effects of aerobic training on cardiopulmonary function and walking economy in people with Parkinson's*

Author	Sample Size	Mean (SD) Age (yr)	Men:Women	Study Design	Intervention	Outcome Measures	Key Findings
Bergen et al.^14^	4 G1; 4 G2	56.8 (6.5)	Not mentioned	Pilot non‐randomized trial	G1: cycling or treadmill walking for 3 times/week for 16 weeks G2: usual activity	CPET	Increased V˙O_2peak_ and achieved workload in the intervention group (from 19.5 to 24.5 mL × kg × min^−1^). Decreased V˙O_2peak_ in the control group (from 15.9 to 14.1 mL × kg × min^−1^).
Bridgewater and Sharpe^18^	13 G1; 13 G2	67.3 (3.9)	9:4	RCT	G1: 12 weeks aerobic exercise (walking) G2: usual physical activity level	CPET	Improvement in exercise test duration and HR in the exercise group The exercise group had a mean (SE) minimum target heart rate of 82.92 (9.98) beats per minute and exercised at an intensity that produced a mean (SE) maximum HR of 121.27 (16.74) beats per minute. Significant group by session interactions between the exercise and control groups in mean CPET duration (*P* = 0.047).
Burini et al.^16^	13 G1; 13 G2	65.2 (6.5)	9:17	RCT	G1: Aerobic training 3 times/week for 7 weeks G2: Qigong exercises 3 times/week for 7 weeks	CPET	G1: mean (SD) V˙O_2peak_ (mL × kg × min^−1^) at baseline =1201.4 (368), and 951 (337) after 7 weeks, within‐group mean difference = 250 (t = 2.3, *P* = 0.04) G2: mean (SD) V˙O_2peak_ (mL × kg × min^−1^) at baseline = 1064.7 (229) and 1158 (307) after 7 weeks (mean difference = −94); within‐group mean difference = −94 (*P* = 0.02) Significant between‐group difference for V˙O_2peak_ (*P* = 0.007).
Corbianco et al.^15^	10 G1; 10 G2	58.8 (3.9)	20:0	RCT	G1: treadmill training 20 minutes per day, 4 days/week for 4 weeks G2: whole body vibration 20 min per day, 4 days/week for 4 weeks		VO_2 peak_ increased in both groups (mL × kg × min^−1^): G1: baseline mean (SD) V˙O_2peak_ (13.46 [4.96]), after 4 weeks (18.55 [1.11]) G2: baseline mean (SD) V˙O_2peak_ (13.22 [6.16]), after 4 weeks (20.70 [1.16]) Between‐group difference for V˙O_2peak_ non‐significant
Fernández‐del‐Olmo et al.^22^	11 G1; 11 G2	58.7 (10)	13:9	RCT	G1: treadmill training 3 times/week for 5 weeks G2: overground training 3 times/weeks for 5 weeks	Overground walking economy	Treadmill training but not overground training reduced overground walking economy (*P* < 0.001 for treadmill training). Mean (SD) change within group (mL × kg × min^−1^): G1 = 15.54 (3.24) G2 = 19.40 (4.78)
Mavrommati et al.^17^	36 G1; 47 G2	67 (8)	61:22	RCT	G2: treadmill, cycle ergometer, cross‐trainer or rowing ergometer 2 times/week for 24 weeks G2: usual activity	CPET	G2 obtained higher maximum values for HR, VO_2 peak_. Mean (SD) V˙O_2peak_ (mL × kg × min^−1^): G1 = 1.66 (2.35), G2 = 1.69 (2.57) Mean (SD) HR _peak_ (beats × min^−1^): (G1 = 136 (114), G2 = 152 (108)) Significant difference between the two groups for V˙O_2peak_ (*P* = 0.008)
Ridgel et al.^20^	22 G1; 22 G2	70.2 (7.9)	19:5	RCT	G1: combined intensive therapy including cycle resistance and aerobic exercises 3 times/week for 12 weeks G2: usual activity	CPET	No significant differences between both groups for cardiovascular variables (resting HR [*P* = 0.59], V˙O_2 max_ [*P* = 0.86])
Schenkman et al.^21^	39 G1; 41 G2; 41 G3	63.4 (11.2)	76:45	RCT	G1: supervised flexibility, balance, and function, 3 days/week for 3 mo G2: supervised aerobic exercise, 3 days/week for 16 mo G3: control (home exercise), single supervised session and then 5–7 days/week for 16 mo at home	Walking economy	Walking economy was improved in the aerobic exercise group but not in the flexibility, balance, and function group or the control group (mean difference = −1.2 mL × kg × min^−1^, 95% CI = −1.9 to −0.5)
Shulman et al., 2012^19^	26 G1; 26 G2; 28 G3	65.8 (10.7)	50:17	RCT	G1: low‐intensity treadmill training, 3 times/week for 3 mo G2: high‐intensity treadmill training, 3 times/week for 3 mo G3: stretching and resistance training, 3 times/week for 3 mo	CPET	Low‐intensity treadmill intervention had the greatest effect in improving gait speed. Both treadmill interventions decreased maximum V˙O_2._ Mean difference (SD) between baseline and post training (mL × kg × min^−1^): Low intensity (1.54 [0.4]) High intensity (1.53 [0.7]) Statistically significant with *P* = 0.003

SD, standard deviation; G1, group 1; G2, group 2; G3, group 3; CPET, cardiopulmonary exercise test; V˙O_2_, oxygen consumption; RCT, randomized controlled trial; HR, heart rate; SE, standard error.

### Effects of Aerobic Training on Cardiopulmonary Function

Seven studies assessed the effects of aerobic training on cardiopulmonary function. Aerobic training (including walking overground, treadmill walking, and stationary cycling) in PwP was reported to improve V˙O_2peak,_ ^14^, ^15^ decrease breathlessness,^16^ increase maximum workload tolerated,^14^ reduce BP,^17^ and increase test duration of the CPET.^17^, ^18^ However, two studies reported a decrease, rather than an increase, in V˙O_2peak_ after aerobic training,^16^, ^19^ and one study reported no significant differences between the exercise group and the usual activity group.^20^

### Frequency and Duration of the Exercise

Frequency of the aerobic training ranged from 2–5 times per week, with exercise program durations ranging from 6–24 weeks. For example, Bergen et al.^14^ investigated the effects of a 3‐times/week exercise program for 16 weeks (n = 4); Bridgewater and Sharpe,^18^ Ridgel et al.^20^ and Shulman et al.^19^ investigated the effects of a 3‐times/week exercise program for 12 weeks (n = 13, n = 24, and n = 67, respectively); and Mavrommati et al.^17^ investigated the effects of a program 2 times/week for 24 weeks (n = 83). Shorter program durations of 3 times/week for 7 weeks (n = 26)^16^ and 4 days/week for 4 weeks (n = 20)^15^ have also been investigated. Although the duration of the aerobic exercise intervention varied in these studies, all of them reported improvement in exercise test outcomes, including HR and V˙O_2_, except two studies.^16^, ^19^

It is not clear if there is a dose–effect relationship. However, Bergen et al.^14^ reported that V˙O_2peak_ significantly improved by 26%, from 19.5 mL × kg × min^−1^ to 24.5 mL × kg × min^−1^, after 16 weeks of exercise. Mavrommati et al.^17^ reported a mean (SD) improvement in V˙O_2_ of 1.66 (0.09) mL × kg × min^−1^, although the intervention included a 24‐week exercise program. Bridgewater et al.^18^ only reported changes in HR, not in V˙O_2_. Two studies^14^, ^23^ did not report the mean or SD or the mean difference for V˙O_2_. Additionally, these studies used different exercise tests (i.e., cycle CPET or treadmill CPET) and different exercise test protocols. Therefore, these results need to be treated with caution because of this heterogeneity.

### Intensity of the Exercise

Only one study^19^ investigated the effects of different exercise intensities on V˙O_2_, and the other six studies investigated only the effects of specific intensities. To study the effects of different intensities of aerobic exercises, Shulman et al.^19^ assessed V˙O_2_ and gait speed after high‐ and low‐intensity treadmill walking training. A high‐intensity treadmill group (n = 34, Hoehn and Yahr [H&Y] stages I–III) started at 40% to 50% of maximal HR and increased up to 70% to 80%. In contrast, the low‐intensity treadmill walking training (n = 33, H&Y stages I–III) started at 20% and increased up to 40% to 50% of maximal HR. Both high‐ and low‐intensity treadmill training improved V˙O_2_, to a similar degree, with a 1.54 mL × kg × min^−1^ increase after low‐intensity and 1.53 1.54 mL × kg × min^−1^ increase after high‐intensity treadmill training. However, Ridgel et al.^20^ reported no change in V˙O_2_ or any other markers of cardiovascular response after moderate intensity aerobic training involving 44 PwP at H&Y stages I–III. It should be noted that this study included a combined aerobic‐strengthening program and was not solely focused on aerobic training. Other studies reported using intensities of 60% to 70%,^14^ 65% to 85%,^18^ 59% to 60%,^16^ and 55% to 85%^17^ maximal HR. Corbianco et al.^15^ used the Borg scale for assessing rate of perceived exertion (RPE) as an intensity to do exercise with score of 13–15 on the 20‐point Borg scale.^15^

### The Effects of Aerobic Exercise on Walking Economy

Walking economy is defined as the energy required to perform at a submaximal walking intensity.^24^ It is measured by the rate of V˙O_2_ per distance during walking.^24^ Only two studies^21^, ^22^ investigated the effect of aerobic training on cardiopulmonary function and walking economy in PwP. Table 3 summarizes the main findings of these two studies.

Schenkman et al.^21^ examined the effect of 30 minutes of aerobic exercise, three times/week for 16 weeks, and reported decrease in oxygen consumption (mean difference = −1.2 mL × kg × min^−1^, 95% CI = −1.9 to −0.5). However, Schenkman's study assessed the energy required to walk on the treadmill, not on an overground surface that is more akin to functional mobility. Only one study^22^ examined the effect of treadmill training and overground walking on overground walking economy in PwP. To achieve this, 22 PwP (H&Y stage I–II) were randomly allocated to an overground walking intervention group and a treadmill training group for 5 weeks. Both groups were required to walk at their preferred speed three times/week for 5 weeks. Results of the study indicated that treadmill training, but not overground training, reduced overground energy expenditure by a mean (SD) of 15.54 (3.24) mL × kg × min^−1^ versus 19.40 (4.78) mL × kg × min^−1^, respectively. This may have been because the controlled speed of the treadmill elicited a higher intensity of exercise for the duration of the activity than overground walking. Although this study used a small sample (n = 22), it is the only study to have assessed overground walking economy in PwP.

## Discussion

This review has shown that only a small number of studies have investigated the effects of aerobic exercise on cardiopulmonary function in Parkinson's, and it has indicated that aerobic exercise could help in improving cardiac fitness. Aerobic exercise is considered to be a cheap, widely accepted self‐managed intervention. Most of the studies included in the review used similar intensities and frequencies recommended by the World Health Organization (WHO) (30–45 minutes of moderate intensity, three times per week).^25^ However, none of these studies investigated the effects of aerobic exercise on pulmonary function. Therefore, future research is recommended to investigate the effects of aerobic exercise on cardiac fitness and pulmonary function in PwP, with an appropriately calculated sample size and different disease severities.

The decrease in walking economy in PwP leads to excess fatigue and, in turn, could affect independence and quality of life.^24^ However, only two studies^21^, ^22^ studied walking economy. Investigating walking economy could help an understanding of whether PwP needs to consume more energy to walk, and how different interventions could affect energy consumption. There is a lack of knowledge of the factors that might affect walking economy. Further studies are needed to determine functional (overground) walking economy, because this might affect functional activity, participation, and overall quality of life in this population.

### Strengths and Limitations of the Review

This is the first systematic review addressing the effects of aerobic exercise on cardiopulmonary function in PwP. The search included relevant studies from the last 40 years and involved screening by two reviewers. However, it was not feasible to do meta‐analysis because of the heterogeneity of the data and the outcome measures, methods, and protocols used in the included studies. Additional data that might have enabled effect size calculation were not made available by the authors.▪

## Conclusion

This review has collated the reported effects of aerobic training on cardiopulmonary function and walking economy in PwP and concludes that aerobic exercise could help in improving cardiac fitness and walking economy. However, no research has been done to investigate the effects of aerobic exercise specifically on pulmonary function using spirometry in PwP. Therefore, further research that may help to determine the effects of aerobic exercise on pulmonary function is warranted.

## Author Roles

(1) Research Project: A. Conception, B. Organization, C. Execution; (2) Statistical Analysis: A. Design, B. Execution, C. Review and Critique; (3) Manuscript: A. Writing of the First Draft, B. Review and Critique.

A.A.: 1A, 1B, 1C, 2A, 2B, 3A, 3B

S.J.L.: 1A, 1B, 3B

J.S.: 1B, 2C, 3B

S.M.H.: 1A, 1B, 2C, 3B

## Disclosures

**Ethical Compliance Statement**: The authors confirm that the approval of an institutional review board and patient consent was not required for this work. We have read the Journal's position on issues involved in ethical publication and affirm that this work is consistent with those guidelines.

**Funding Sources and Conflicts of Interest**: A.A. is supported by a scholarship from Isra University. The authors declare that there are no conflicts of interest relevant to this work.

**Financial Disclosures for previous 12 months:** No specific funding was received for this work.
